# Frequency, prognosis and treatment modalities of newly diagnosed small bowel cancer with liver metastases

**DOI:** 10.1186/s12876-020-01487-6

**Published:** 2020-10-15

**Authors:** Xiaorong Ye, Lifu Wang, Yongjun Xing, Chengjun Song

**Affiliations:** Department of Trauma Surgery, The Lishui People’s Hospital, 15 Dazhong Street, Lishui, 323000 Zhejiang People’s Republic of China

**Keywords:** Small bowel cancer, Liver metastases, Frequency, Prognosis, Treatment modality

## Abstract

**Background:**

Population-based analysis for the liver metastases of small bowel cancer is currently lacking. This study aimed to analyze the frequency, prognosis and treatment modalities for newly diagnosed small bowel cancer patients with liver metastases.

**Methods:**

Patients with small bowel cancer diagnosed from 2010 to 2015 were extracted from the Surveillance, Epidemiology, and End Results (SEER) database. Binary logistic regression analysis was performed to determine predictors for the presence of liver metastases at diagnosis. Kaplan–Meier method and Cox regression analyses were performed for survival analyses.

**Results:**

A total of 1461 small bowel cancer patients with liver metastases at initial diagnosis were identified, representing 16.5% of the entire set and 63.9% of the subset with metastatic disease to any distant site. Primary tumor with poorer histological type, larger tumor size, later N staging, more extrahepatic metastatic sites, and tumor on lower part of small intestine had increased propensity of developing liver metastases. The combined diagnostic model exhibited acceptable diagnostic efficiency with AUC value equal to 0.749. Patients with liver metastases had significant poorer survival (*P* < 0.001) than those without liver metastases. In addition, combination of surgery and chemotherapy (HR = 0.27, *P* < 0.001) conferred the optimal survival for patients with adenocarcinoma, while the optimal treatment options for NEC and GIST seemed to be surgery alone (HR = 0.24, *P* < 0.001) and chemotherapy alone (HR = 0.08, *P* = 0.022), respectively.

**Conclusions:**

The combined predictor had a good ability to predict the presence of liver metastases. In addition, those patients with different histologic types should be treated with distinct therapeutic strategy for obtaining optimal survival.

## Background

Small bowel cancer represents a heterogenous group of malignancies, which occurs mainly in the three anatomical segments of the small intestine, including duodenum, jejunum and ileum [1, 2]. Although the most common histological type are adenocarcinoma, carcinoids, sarcomas and lymphomas, more than forty different histological subtypes have been described recently [3]. In contrast to large bowel cancer, the incidence of small bowel cancer has been increasing. Based on the latest cancer statistic report, an estimated 10,470 new cases of small bowel cancer are expected to be diagnosed in 2018 in the United States nationally, with 1450 deaths caused by this disease [4]. Although rare, small bowel cancers have an incidence rate comparable to testicular cancer, chronic myeloid leukaemia, Hodgkin disease and anal cancer [4].

A certain proportion of small bowel cancer patients presented with evidence of distant metastases at the initial diagnosis, wherein liver exhibited the most common metastatic organ [5, 6]. Evidently, the presence of liver metastases served as an important predictor for worse prognosis of small bowel cancer [6, 7], which may due to the increasing tumor burden and impairment of vital organ function caused by disease progression. Owing to the rarity of small bowel cancer with liver metastases, a population-based study regarding to the frequency as well as the prognosis for those population was still lacking. Meanwhile, due to the rarity and non-specific presentation, a major part of patients were diagnosed with advanced stage [8, 9], causing a controversial therapeutic strategy, especially for those patients with metastasis disease [10]. Although it is our belief that metastasis cancer (IV stage) is incurable, a few researches had showed that hepatic resection in patients with oligometastatic liver disease might improve survival [11, 12]. Moreover, palliative surgery also might be necessary in selected cases for relief of bowel obstruction [13, 14]. Several previous studies have indicated that adjuvant chemotherapy was associated with improved survival for patients with small bowel cancer [15, 16]. However, other retrospective studies did not demonstrate survival benefit of adjuvant therapy [5, 17]. Therefore, a large population based study concentrating on describing epidemiologic characteristics, prognosis and optimal treatment modalities of small bowel cancer patients with liver metastases was urgently needed.

In current study, we investigated the incidence and predictors for liver metastases among patients with small bowel cancer by using the SEER database. The prognostic factors associated with the survival of patients with liver metastases were subsequently studied. Furthermore, we also attempted to explore the optimal treatment modalities based on the survival data of small bowel cancer patients with liver metastases.

## Methods

### Database and case selection

**Fig. 1:**
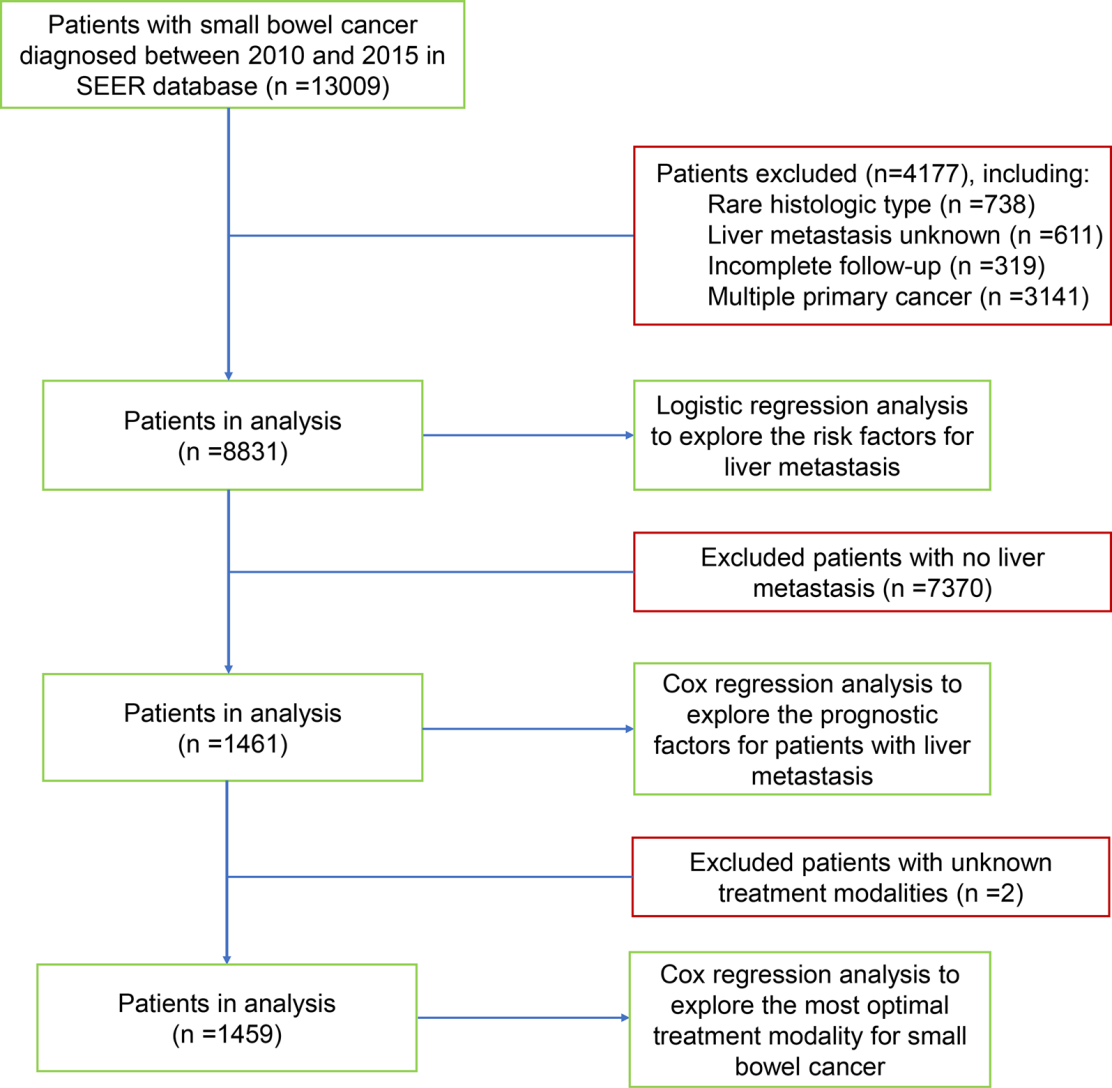
Flowchart of data selection and grouping. *SEER* surveillance, epidemiology, and end results program

Data was obtained from the recently released SEER database [Incidence-SEER 18 Regs Custom Data (with additional treatment fields), Nov 2018 Sub], which collected cancer data that covers about 28 percent of the United States population [4]. We used SEER*Stat software version 8.3.6 (National Cancer Institute, USA) to access the data from SEER database. A total of 13,009 patients with small bowel cancer (Site recode International Classification of Diseases for Oncology-3 (ICD-O-3)/WHO 2008: small intestine) with malignant behavior who were diagnosed from 2010 to 2015 were extracted from the database. Only patients with one primary cancer were included in this study. Moreover, patients with incomplete follow-up, unknown liver metastasis information, and histological type other than adenocarcinoma, neuroendocrine tumors (NETs) and gastrointestinal stromal tumors (GISTs) were excluded. Since it was difficult to accurately differentiate NET or NEC in SEER program based on the recent 2019 WHO classification, NEC and NET were classified into one category as NETs in this study. A total of 8831 eligible small bowel cancer patients were subjected to binary logistic regression analysis to explore the risk factors for the presence of liver metastases. Then, 1461 eligible small bowel cancer patients with liver metastases were selected to perform univariate and multivariate Cox regression analysis for the purpose of exploring the prognostic factors. Furthermore, after exclusion of 2 patients with unknown treatment modalities (surgery, chemotherapy and radiotherapy), we performed multivariate Cox regression analysis to explore the optimal treatment option with greatest survival benefits for small bowel cancer patients who had liver metastases. The flowchart of case selection was shown in Fig. 1.

### Covariates

The analysis involved multiple variables including demographic characteristics (year of diagnosis, age at diagnosis, marital status, insurance status, race, and gender), disease characteristics (primary site, histologic grade, tumor size, liver metastases, AJCC T and N stage, and numbers of extrahepatic metastatic sites), and treatment characteristics (surgery, chemotherapy, and radiotherapy). Specially, the continuous variables, including age at diagnosis and tumor size, were transformed into categorical variables. Marital status including single, divorced, widowed, separated and domestic partner were classified into unmarried. Insurance recode including insured and any medicaid were classified into insured. Vital status record recode and cause-specific death classification were utilized to define the main outcomes including overall survival (OS) and cancer-specific survival (CSS).

### Statistical analysis

Descriptive statistic was utilized to summarize the baseline characteristics of patients with liver metastases among the entire set, or among the patients with metastatic disease to any distant site at the time of cancer diagnosis. Clinicopathologic characteristics between patients with or without liver metastases were compared using Pearson chi-square tests. Univariate and multivariable logistic regression were performed to determine predictors of the presence of liver metastases at diagnosis. Kaplan–Meier plot and log-rank test were used to compare differences of OS and CSS between patients with or without liver metastases. The univariate and multivariate Cox regression analysis were used to estimate hazard ratio (HR) for OS and CSS. In addition, Cox regression model were also conducted to put up multiple comparison across different histological types of small bowel cancer for survival benefit of different treatment modalities by setting up different reference.

Descriptive statistic, Pearson Chi-square test, binary logistic regression model, and Cox proportional hazards model were performed using SPSS 24.0 (IBM Corp). Kaplan–Meier plot and log-rank test were plotted or conducted by using R software version 3.6.0. A 2-sided *P* value of < 0.05 was considered as statistical significance unless otherwise stated.

## Results

### Demographic characteristics of patients

Based on the inclusion criteria, a total of 8831 patients with small bowel cancer were extracted from the SEER database, including 2457 (27.8%) adenocarcinoma, 5406 neuroendocrine tumors (NETs) (61.2%), and 968 gastrointestinal stromal tumors (GISTs) (11%). Among the 2285 (25.9%) patients who had synchronous metastases at the time of diagnosis, a total of 1461 (63.9%) patients presented with synchronous liver metastases, which consisted of 506 adenocarcinoma, 863 NETs, and 92 GISTs. The detailed demographic and clinical characteristics of those patients with small bowel cancer were summarized in Table 1.

**Table 1:** Clinical characteristics of small bowel cancer patients with liver metastases at diagnosis

Variable	Patients, no			Proportion of liver metastases, %		Survival among patients with liver metastases, median (IQR), mo
	With small bowel cancer (n = 8831)	With metastatic disease (n = 2285)	With liver metastases (n = 1461)	Among entire cohort	Among subset with metastatic disease	
Age						
< 40	369	86	64	17.34	74.42	38.3 (18.0–59.0)
40–59	2998	782	509	16.98	65.09	38.7 (20.0–57.0)
60–79	4371	1136	727	16.63	64.00	33.5 (14.0–51.0)
≥ 80	1093	281	161	14.73	57.30	23.4 (2.0–40.0)
Race						
Black	1485	365	241	16.23	66.03	32.8 (13.0–51.0)
White	6791	1806	1148	16.90	63.57	34.7 (15.0–54.0)
Others^a^	465	110	69	14.84	62.73	31.7 (13.0–49.5)
Unknown	90	4	3	3.33	75.00	32.3 (16.0–50.3)
Gender						
Male	4553	1179	765	16.80	64.89	34.0 (14.5–52.0)
Female	4278	1106	696	16.27	62.93	34.5 (14.0–53.0)
Insurance status						
No	263	78	52	19.77	66.67	35.3 (12.0–59.0)
Yes	8373	2179	1390	16.60	63.79	34.2 (15.0–53.0)
Unknown	195	28	19	9.74	67.86	33.2 (14.0–51.0)
Marital status						
Unmarried	3222	842	529	16.42	62.83	31.5 (12.0–50.0)
Married	5089	1339	859	16.88	64.15	36.0 (16.0–55.0)
Unknown	520	104	73	14.04	70.19	34.2 (16.0–53.8)
Primary site						
Duodenum	3201	667	438	13.68	65.67	28.3 (8.0–46.0)
Jejunum	848	232	111	13.09	47.85	35.6 (17.0–54.0)
Ileum	2572	682	458	17.81	67.16	39.5 (21.0–57.0)
Other site^b^	141	27	16	11.35	59.26	36.0 (16.0–56.5)
Unknown	2069	677	438	21.17	64.70	36.1 (17.0–55.0)
Grade						
I	3704	691	464	12.53	67.15	37.9 (20.0–55.0)
II	1908	483	290	15.20	60.04	30.9 (13.0–48.0)
III	849	332	186	21.91	56.02	18.5 (3.0–27.0)
IV	125	77	27	21.60	35.06	29.9 (14.0–46.0)
Unknown	2245	1514	494	22.00	32.63	37.2 (14.0–60.5)
Histologic type						
Adenocarcinoma	2457	886	506	20.59	57.11	20.0 (3.0–29.0)
NEC	5406	1205	863	15.96	71.62	39.5 (21.0–57.0)
GISS	968	194	92	9.50	47.42	41.1 (21.0–60.0)
T stage						
T1	1382	157	98	7.09	62.42	33.2 (14.0–52.0)
T2	1306	157	114	8.73	72.61	40.8 (22.0–60.0)
T3	2767	627	418	15.11	66.67	38.4 (19.0–57.0)
T4	2128	803	427	20.07	53.18	29.7 (11.0–46.0)
Unknown	1248	541	404	32.37	74.68	27.1 (4.25–46.0)
Tumor size, cm						
0–1	1518	86	52	3.43	60.47	38.3 (20.0–56.0)
1–2	1897	413	267	14.07	64.65	38.9 (20.0–57.0)
2–5	2523	751	488	19.34	64.98	34.7 (15.0–53.0)
> 5	1325	347	176	13.28	50.72	33.8 (14.0–52.0)
Unknown	1568	688	478	30.48	69.48	24.3 (16.0–42.0)
N stage						
N0	4732	859	531	11.22	61.82	34.5 (14.0–54.0)
N1	3430	1096	728	21.22	66.42	36.7 (18.0–55.0)
N2	315	115	51	16.19	44.35	20.9 (7.0–29.0)
Unknown	354	215	151	42.66	70.23	18.9 (2.0–29.0)
M stage						
M0	6546	6546	0	0.00	0.00	37.1 (18.0–56.0)
M1	2285	2285	1461	63.94	63.94	26.1 (6.0–42.0)
Extrahepatic metastatic sites to bone, lung, and brain, No						
0	8488	1953	1255	14.79	64.26	35.0 (15.0–54.0)
1	243	243	142	58.44	58.44	14.1 (2.0–21.0)
2	16	16	9	56.30	56.30	9.5 (2.3–14.8)
Unknown	84	73	55	65.48	75.34	20.6 (1.3–36.8)
Surgery						
No	1865	946	674	36.14	71.24	19.7 (3.0–30.0)
Yes	6935	1335	785	11.32	58.80	38.1 (19.0–56.0)
Unknown	31	4	2	6.45	50.00	36.7 (14.0–56.0)
Radiotherapy						
No	8563	2177	1387	16.20	63.71	34.5 (15.0–53.0)
Yes	268	108	74	27.61	68.52	24.3 (9.0–37.8)
Chemotherapy						
No	6820	1406	918	13.46	65.29	35.6 (16.0–55.0)
Yes	2011	879	543	27.00	61.77	29.7 (12.0–44.0)

*IQR* interquartile range, *CI* confidence interval, *NEC* neuroendocrine carcinoma, *GISS* gastrointestinal stromal sarcoma

^a^Asian and American Indians

^b^Meckels diverticulum, and overlapping lesion of small intestine

The baseline characteristics between patients with or without liver metastases were compared by Chi-square test (Additional file 1: Table S1). As shown in Additional file 1: Table S1, a significant difference could be found in constituent ratio of race, insurance status, tumor primary site, grade, histological type, AJCC T and N stage, tumor size, number of extrahepatic metastatic sites, surgery, radiotherapy, and chemotherapy. Patients without liver metastases had higher proportion of duodenum (37.5% vs 30.0%), jejunum tumor (10.0% vs 7.6%), but less ileum tumor (28.7% vs 31.3%) as compared with patients diagnosed with liver metastases. Patients who had liver metastases tended to have higher histological grade, later AJCC T and N stage, and more numbers of extrahepatic metastatic sites than patients without liver metastases. In addition, patients with liver metastasis had received more adjuvant therapy, such as chemotherapy (37.2% vs 19.9%) or radiotherapy (5.1% vs 2.6%), but less surgical treatment (53.7% vs 83.4%) than patients without liver metastases.

### Predictors for the presence of liver metastases

In order to identify the possible predictors associated with occurrence of liver metastases, univariate and multivariate binary logistic regression analysis were performed (Table 2). The multivariate analysis showed that jejunum (vs duodenum; OR = 1.38; 95% CI [1.07–1.78]; *P* = 0.012), ileum (vs duodenum; OR = 2.06; 95% CI [1.70–2.50]; *P* < 0.001), grade III (vs grade I; OR = 1.54; 95% CI [1.21–1.96]; *P* = 0.001), grade IV (vs grade I; OR = 2.54; 95% CI [1.56–4.12]; *P* < 0.001), tumor size 1–2 cm (vs tumor size 0–1 cm; OR = 3.83; 95% CI [2.66–5.52]; *P* < 0.001), tumor size 2–5 cm (vs tumor size 0–1 cm; OR = 5.53; 95% CI [3.85–7.94]; *P* < 0.001), tumor size > 5 cm (vs tumor size 0–1 cm; OR = 4.38; 95% CI [2.91–6.59]; *P* < 0.001), N1 (vs N0; OR = 1.87; 95% CI [1.60–2.18]; *P* < 0.001), 1 extrahepatic metastatic site (vs 0 extrahepatic metastatic site; OR = 5.14; 95% CI [3.86–6.84]; *P* < 0.001), 2 extrahepatic metastatic site (vs 0 extrahepatic metastatic site; OR = 4.35; 95% CI [1.53–12.3]; *P* = 0.006) were significantly associated with greater odds of having liver metastases at initial diagnosis. On the contrary, gastrointestinal stromal tumor (GIST) (vs adenocarcinoma; OR = 0.56; 95% CI [0.41–0.75]; *P* < 0.001) were significantly associated with lower odds of liver metastases at diagnosis. Taken together, these data suggested that small bowel cancer patients with factors like lower part of small intestine, poorer histological grade, larger tumor size, later N staging, and presence of more extrahepatic metastatic sites showed an increased propensity for developing liver metastases.

**Table 2:** Factors associated with the presence of liver metastases at diagnosis of small bowel cancer

Variables	Univariate logistic model		Multivariate logistic model	
	OR (95% CI)	*P* value	OR (95% CI)	*P* value
Age				
< 40	Reference		NA	
40–59	0.98 (0.73–1.30)	0.860		
60–79	0.95 (0.72–1.26)	0.725		
≥ 80	0.82 (0.60–1.13)	0.229		
Race				
Black	Reference		NA	
White	1.05 (0.90–1.22)	0.528		
Others^a^	0.90 (0.67–1.20)	0.474		
Unknown	0.18 (0.06–0.57)	0.004		
Gender				
Male	Reference		NA	
Female	0.96 (0.86–1.08)	0.501		
Insurance status				
No	Reference		NA	
Yes	0.81 (0.59–1.10)	0.175		
Unknown	0.44 (0.25–0.77)	0.004		
Marital status				
Unmarried	Reference		NA	
Married	1.03 (0.92–1.16)	0.583		
Unknown	0.83 (0.64–1.08)	0.171		
Primary site				
Duodenum	Reference		Reference	
Jejunum	0.95 (0.76–1.19)	0.654	1.38 (1.07–1.78)	0.012
Ileum	1.37 (1.19–1.58)	< 0.001	2.06 (1.70–2.50)	< 0.001
Other site^b^	0.81 (0.48–1.37)	0.429	1.18 (0.66–2.09)	0.580
Unknown	1.69 (1.46–1.96)	< 0.001	2.28 (1.89–2.74)	< 0.001
Grade				
I	Reference		Reference	
II	1.25 (1.07–1.47)	0.005	1.11 (0.92–1.34)	0.263
III	1.96 (1.62–2.37)	< 0.001	1.54 (1.21–1.96)	0.001
IV	1.92 (1.24–2.98)	0.003	2.54 (1.56–4.12)	< 0.001
Unknown	1.97 (1.71–2.27)	< 0.001	1.69 (1.43–2.00)	< 0.001
Histologic type				
Adenocarcinoma	Reference		Reference	
NEC	0.73 (0.65–0.83)	< 0.001	0.98 (0.80–1.18)	0.800
GISS	0.41 (0.32–0.51)	< 0.001	0.56 (0.41–0.75)	< 0.001
T stage				
T1	Reference		Reference	
T2	1.25 (0.95–1.66)	0.116	0.57 (0.40–0.80)	0.001
T3	2.33 (0.95–1.66)	< 0.001	0.71(0.52–0.97)	0.029
T4	3.29 (1.85–2.94)	< 0.001	0.94 (0.69–1.28)	0.679
Unknown	6.27 (4.95–7.95)	< 0.001	1.62 (1.19–2.21)	0.002
Tumor size, cm				
0–1	Reference		Reference	
1–2	4.62 (3.40–6.27)	< 0.001	3.83 (2.66–5.52)	< 0.001
2–5	6.76 (5.04–9.07)	< 0.001	5.53 (3.85–7.94)	< 0.001
> 5	4.32 (3.14–5.94)	< 0.001	4.38 (2.91–6.59)	< 0.001
Unknown	12.4 (9.19–16.6)	< 0.001	6.75 (4.67–9.73)	< 0.001
N stage				
N0	Reference		Reference	
N1	2.13 (1.89–2.41)	< 0.001	1.87 (1.60–2.18)	< 0.001
N2	1.53 (1.12–2.09)	0.008	1.19 (0.84–1.69)	0.339
Unknown	5.89 (4.68–7.40)	< 0.001	2.12 (1.62–2.77)	< 0.001
Extrahepatic metastatic sites to bone, lung, and brain, No				
0	Reference		Reference	
1	8.10 (6.24–10.5)	< 0.001	5.14 (3.86–6.84)	< 0.001
2	7.41 (2.76–19.9)	< 0.001	4.35 (1.53–12.3)	0.006
Unknown	10.9 (6.94–17.2)	< 0.001	6.29 (3.89–10.2)	< 0.001

*CI* confidence interval, *OR* odds ratio, *NEC* neuroendocrine carcinoma, *GISS* gastrointestinal stromal sarcoma

^a^Asian and American Indians

^b^Meckels diverticulum, and overlapping lesion of small intestine

**Fig. 2:**
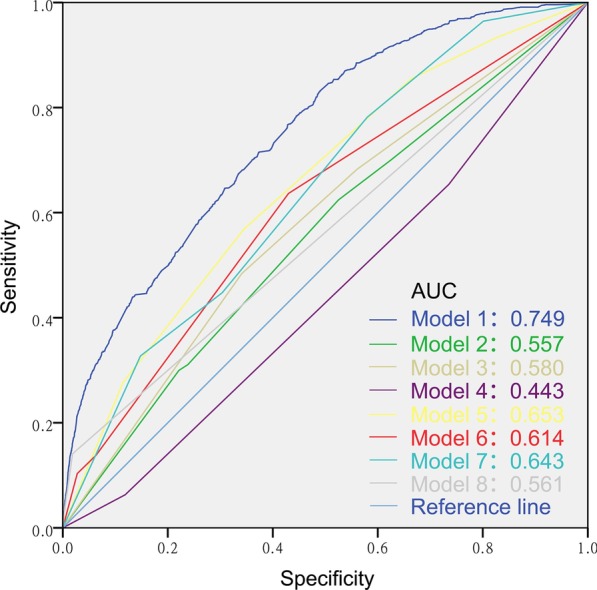
ROC curves to predict the presence of liver metastases in the entire set stratified by model 1 (combined predictors, AUC: 0.749), model 2 (primary site, AUC: 0.557), model 3 (grade, AUC: 0.580), model 4 (histologic type, AUC: 0.443), model 5 (AJCC T stage, AUC: 0.653), model 6 (AJCC N stage, AUC: 0.614), model 7 (tumor size, AUC: 0.643) and model 8 (number of extrahepatic metastatic sites, AUC: 0.561)

Subsequently, the ROC curve was plotted to evaluate the predicting performance (Fig. 2). The multivariable logistic model, which incorporated seven significant variables, exhibited dramatically higher AUC (AUC: 0.749; 95% CI [0.735–0.762]; *P* < 0.001) value than separate variables, including primary site (AUC: 0.557; 95% CI [0.541–0.574]), grade (AUC: 0.580; 95% CI [0.564–0.596]), histologic type (AUC: 0.443; 95% CI [0.428–0.459]), AJCC T stage (AUC: 0.653; 95% CI [0.638–0.668]), tumor size (AUC: 0.643; 95% CI [0.628–0.657]), AJCC N stage (AUC: 0.614; 95% CI [0.598–0.630]), and number of extrahepatic metastatic sites (AUC: 0.561; 95% CI [0.544–0.578]). These results suggested that our model had a good performance for discriminating patients prone to occur liver metastases.

### Survival analysis of small bowl cancer patients with liver metastases

**Fig. 3:**
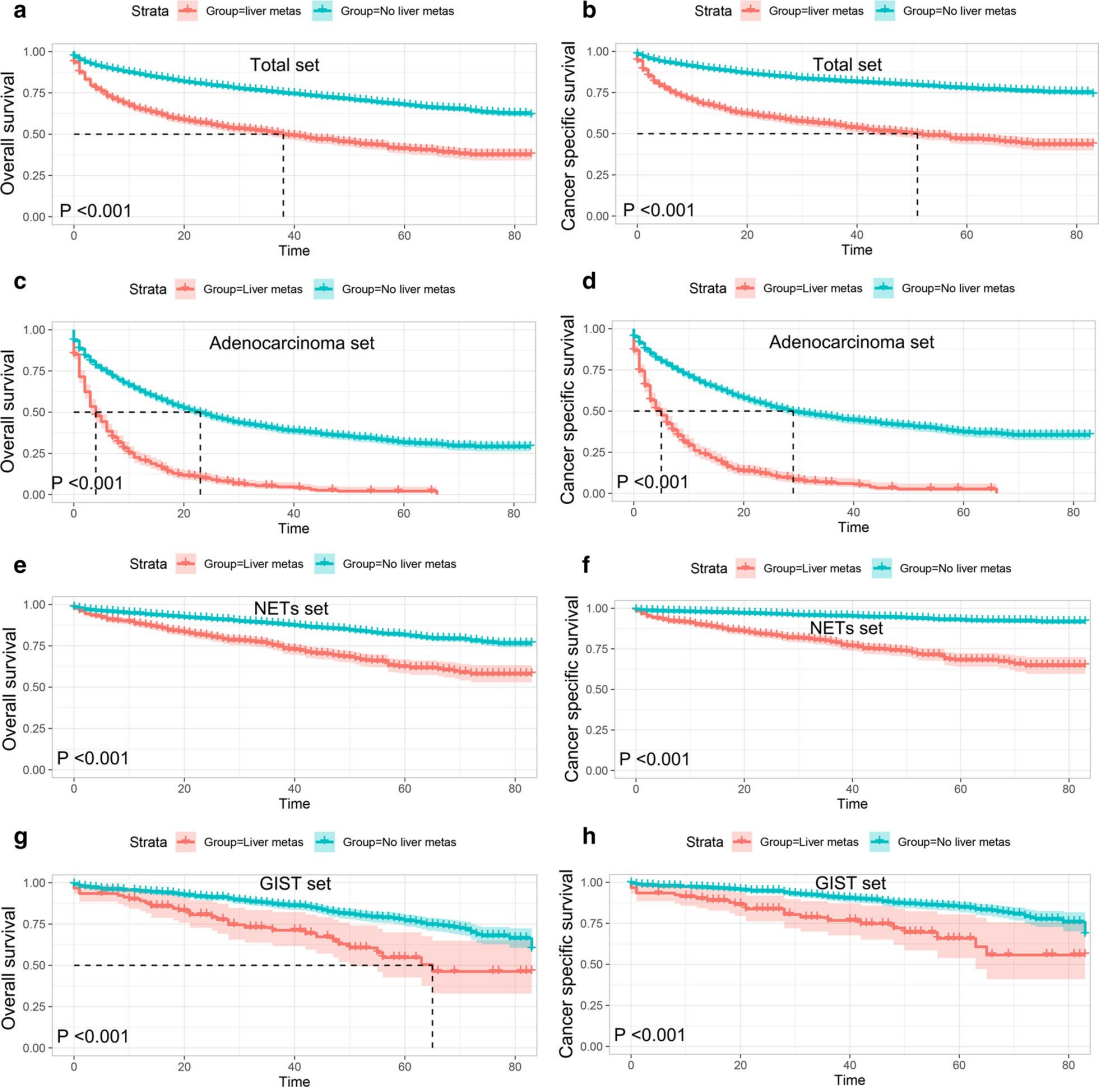
Overall survival (OS) (**a**) and Cancer-specific survival (CSS) (**b**) curves plotted by Kaplan–Meier method for patients with or without liver metastases in total set; OS (**c**) and CSS (**d**) for patients with or without liver metastases in adenocarcinoma set; OS (**e**) and CSS (**f**) for patients with or without liver metastases in NETs set; OS (**g**) and CSS (**h**) for patients with or without liver metastases in GIST set

The Kaplan–Meier method was used to compare the OS and CSS between patients with or without liver metastases. As shown in Fig. 3a, b, patients with liver metastases had significantly poorer OS (*P* < 0.001) and CSS (*P* < 0.001) than those without liver metastases in the total set. Similar trends were also seen in all subsets, including adenocarcinoma, NETs and GISTs patients (Fig. 3c–h).

Of all the 1461 patients who had liver metastases, a total of 709 (48.5%) patients were dead at the time of last follow-up, among which 660 (45.2%) patients were dead directly from small bowel cancer. Subsequently, among the subsets (adenocarcinoma, NETs and GISTs) with liver metastases, univariate and multivariate Cox regression analyses were separately performed to identify factors which significantly associated with OS and CSS. Additional file 2: Table S2, Additional file 3: Table S3, Additional file 4: Table S4 showed univariate analysis for OS and CSS among patients with adenocarcinoma, NETs and GISTs, respectively. The multivariable Cox analysis for adenocarcinoma set showed that tumors occurred in jejunum (vs. duodenum) was significantly associated with an increased OS (HR = 0.65; 95% CI [0.46–0.92]; *P* = 0.014) and CSS (HR = 0.61; 95% CI [0.43–0.88]; *P* = 0.008) (Table 3). Moreover, as shown in Table 4, the multivariate analysis for NETs indicated that patients with age at 60–79 (vs age < 40; HR = 6.86; 95% CI [2.18–21.6]; *P* = 0.001), ≥ 80 (vs age < 40; HR = 10.1; 95% CI [3.09–33.3]; *P* < 0.001), grade III (vs grade I; HR = 5.73; 95% CI [3.22–10.2]; *P* < 0.001), grade IV (vs grade I; HR = 8.46; 95% CI [3.81–18.8]; *P* < 0.001), or 1 extrahepatic metastatic site (vs 0 extrahepatic metastatic site; HR = 2.07; 95% CI [1.36–3.15]; *P* = 0.001) were significantly associated with decreased OS. On the contrary, patients who had tumors occurred in jejunum (vs duodenum; HR = 0.39; 95% CI [0.17–0.91]; *P* = 0.030), or ileum (vs duodenum; HR = 0.43; 95% CI [0.28–0.67]; *P* < 0.001) were significantly associated with increased OS. Similar result was also presented for CSS in Table 4. However, no significant prognostic factor was found among GIST patients who had liver metastases (Additional file 4: Table S4).

**Table 3:** Multivariate analysis for overall survival (OS) and cancer-specific survival (CSS) among patients with small bowel adenocarcinoma who had liver metastasis

Variables	OS		CSS	
	HR (95% CI)	*P* value	HR (95% CI)	*P* value
Age				
< 40	Reference		Reference	
40–59	0.81 (0.48–1.36)	0.417	0.77 (0.45–1.29)	0.312
60–79	0.95 (0.58–1.58)	0.854	0.83 (0.50–1.37)	0.461
≥ 80	1.52 (0.89–2.62)	0.129	1.39 (0.80–2.40)	0.241
Race				
Black	Reference		Reference	
White	1.14 (0.90–1.45)	0.262	1.15 (0.90–1.48)	0.255
Others^a^	1.00 (0.66–1.52)	0.988	1.09 (0.71–1.67)	0.687
Primary site				
Duodenum	Reference		Reference	
Jejunum	0.65 (0.46–0.92)	0.014	0.61 (0.43–0.88)	0.008
Ileum	0.81 (0.55–1.21)	0.307	0.86 (0.57–1.28)	0.453
Other site^b^	0.42 (0.15–1.15)	0.090	0.46 (0.17–1.27)	0.133
Unknown	0.96 (0.72–1.27)	0.774	0.97 (0.72–1.29)	0.810
T stage				
T1	Reference		Reference	
T2	1.36 (0.62–2.98)	0.445	1.52 (0.69–3.35)	0.299
T3	0.77 (0.53–1.11)	0.165	0.75 (0.51–1.11)	0.155
T4	1.02 (0.76–1.37)	0.888	1.02 (0.75–1.38)	0.913
Unknown	1.30 (0.99–1.70)	0.061	1.37 (1.03–1.82)	0.031
N stage				
N0	Reference		Reference	
N1	0.93 (0.74–1.16)	0.516	0.94 (0.75–1.19)	0.600
N2	0.93 (0.66–1.32)	0.686	0.98 (0.68–1.40)	0.895
Unknown	0.96 (0.73–1.27)	0.793	0.93 (0.69–1.24)	0.604

*CI* confidence interval, *HR* hazard ratio

^a^Asian and American Indians

^b^Meckels diverticulum, and overlapping lesion of small intestine

**Table 4:** Multivariate analysis for overall survival (OS) and cancer-specific survival (CSS) among patients with small bowel neuroendocrine tumors (NETs) who had liver metastasis

Variables	OS		CSS	
	HR (95% CI)	*P* value	HR (95% CI)	*P* value
Age				
< 40	Reference		Reference	
40–59	2.96 (0.93–9.47)	0.067	3.71 (0.90–15.3)	0.070
60–79	6.86 (2.18–21.6)	0.001	8.70 (2.14–35.3)	0.002
≥ 80	10.1 (3.09–33.3)	< 0.001	11.8 (2.78–50.2)	0.001
Marital status				
Unmarried	Reference		Reference	
Married	0.82 (0.63–1.08)	0.153	0.82 (0.61–1.11)	0.203
Unknown	0.81 (0.45–1.47)	0.490	0.83 (0.44–1.59)	0.574
Primary site				
Duodenum	Reference		Reference	
Jejunum	0.39 (0.17–0.91)	0.030	0.37 (0.15–0.92)	0.032
Ileum	0.43 (0.28–0.67)	< 0.001	0.37 (0.23–0.59)	< 0.001
Other site^a^	0.47 (0.11–2.00)	0.309	0.26 (0.04–1.95)	0.191
Unknown	0.54 (0.35–0.82)	0.004	0.47 (0.30–0.73)	0.001
Grade				
I	Reference		Reference	
II	1.14 (0.75–1.74)	0.540	1.13 (0.70–1.82)	0.621
III	5.73 (3.22–10.2)	< 0.001	5.32 (2.81–10.1)	< 0.001
IV	8.46 (3.81–18.8)	< 0.001	10.1 (4.50–22.8)	< 0.001
Unknown	1.98 (1.46–2.70)	< 0.001	1.97 (1.40–2.78)	< 0.001
N stage				
N0	Reference		Reference	
N1	0.76 (0.57–1.02)	0.066	0.76 (0.55–1.05)	0.098
N2	0.78 (0.57–1.06)	0.107	0.73 (0.52–1.03)	0.070
Unknown	1.20 (0.79–1.83)	0.382	1.31 (0.84–2.05)	0.235
Extrahepatic metastatic sites to bone, lung, and brain, No				
0	Reference		Reference	
1	2.07 (1.36–3.15)	0.001	2.32 (1.49–3.61)	< 0.001
2	1.13 (0.73–1.74)	0.590	0.99 (0.39–2.42)	0.961
Unknown	0.63 (0.27–1.46)	0.283	0.52 (0.30–1.84)	0.743

*CI* confidence interval, *HR* hazard ratio

^a^Meckels diverticulum, and overlapping lesion of small intestine

### Associations of treatment modality and survival outcomes

**Fig. 4:**
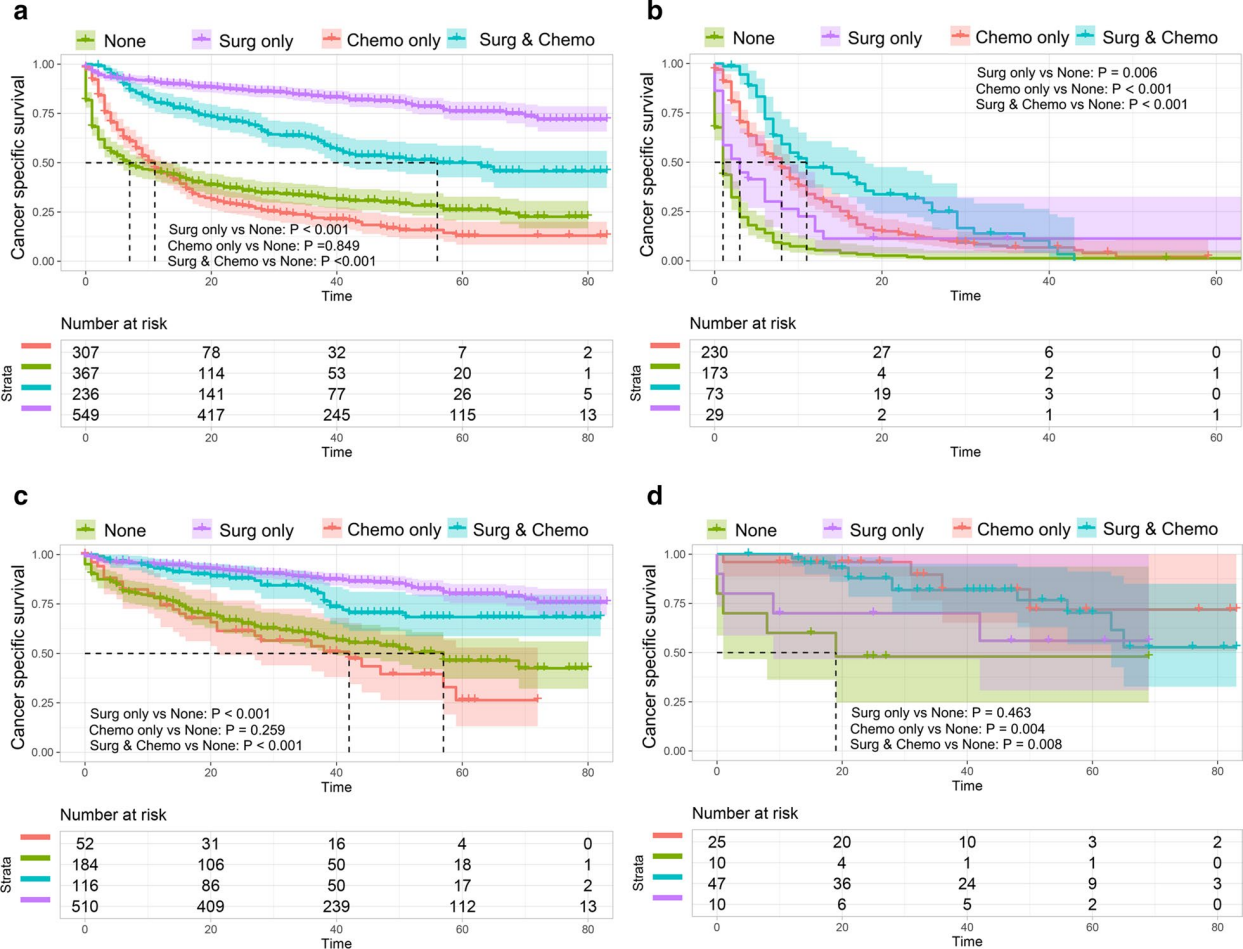
Cancer-specific survival (CSS) for different histological types of small bowel cancer in the entire set (**a**), adenocarcinoma (**b**), Neuroendocrine carcinoma (NEC) (**c**), and Gastrointestinal stromal sarcoma (GISS) (**d**). *P* values were compared using log-rank test

In order to better understand the survival benefit of various treatment modalities, the prognosis of small bowel cancer patients with liver metastases who had received different treatment modalities were compared (Fig. 4). In total set, patients who received surgery (*P* < 0.001) or combination of surgery and chemotherapy (*P* < 0.001) had significantly favorable prognosis as compared with those who received no treatment (Fig. 4a). However, it seems that patients could not benefit from chemotherapy alone (Fig. 4a). For the adenocarcinoma set, all of the treatment modalities significantly increased patients’ CSS (*P* < 0.05) (Fig. 4b). For the NETs set, similar result was obtained to the total set (Fig. 4c). However, for the GISTs set, only chemotherapy alone significantly increased the survival rate as compared with no treatment (Fig. 4d).

Subsequently, multivariate Cox analysis was utilized to unveil the optimal treatment modality for small bowel cancer patients with different histological type. As shown in Table 5, patients with adenocarcinoma could benefit from chemotherapy alone (HR = 0.35, 95% CI [0.27–0.44], *P* < 0.001) or surgery plus chemotherapy (HR = 0.27, 95% CI [0.18–0.42], *P* < 0.001) when compared with no treatment. By setting different reference, surgery plus chemotherapy seemed to be the best therapeutic option (Surgery & chemotherapy vs surgery: HR = 0.37, 95% CI [0.22–0.65], *P* < 0.001; Surgery & chemotherapy vs chemotherapy: HR = 0.67, 95% CI [0.42–1.06], *P* = 0.089). For patients with NETs, treatment modalities including surgery (HR = 0.24, 95% CI [0.14–0.43], *P* < 0.001) and surgery & chemotherapy (HR = 0.39, 95% CI [0.20–0.73], *P* = 0.004) could provide survival benefit when compared with no treatment, and surgery only seemed to be the best therapeutic option (Surgery & chemotherapy vs surgery: HR = 1.48, 95% CI [0.91–2.41], *P* = 0.112; chemotherapy vs surgery: HR = 2.81, 95% CI [1.41–5.63], *P* = 0.003) (Table 5). Moreover, patients with GISTs could only benefit from chemotherapy alone (HR = 0.08, 95% CI [0.01–0.69], *P* = 0.022) (Table 5).

**Table 5:** Association of cancer-specific survival with treatment modality

	Model 1		Model 2		Model 3	
	HR (95% CI)	*P* value	HR (95% CI)	*P* value	HR (95% CI)	*P* value
Part I: univariate analysis						
Small bowel cancer						
None	Ref					
Surgery only	0.15 (0.12–0.19)	< 0.001	Ref			
Chemo only	1.04 (0.87–1.25)	0.681	7.87 (6.18–10.0)	< 0.001	Ref	
Surgery + chemo	0.37 (0.29–0.47)	< 0.001	2.55 (1.93–3.38)	< 0.001	0.32 (0.25–0.41)	< 0.001
Adenocarcinoma						
None	Ref					
Surgery only	0.50 (0.33–0.76)	0.001	Ref			
Chemo only	0.35 (0.28–0.44)	< 0.001	0.70 (0.46–1.07)	0.097	Ref	
Surgery + chemo	0.24 (0.18–0.33)	< 0.001	0.47 (0.29–0.75)	0.002	0.66 (0.49–0.89)	0.006
Neuroendocrine carcinoma (NEC)						
None	Ref					
Surgery only	0.25 (0.18–0.35)	< 0.001	Ref			
Chemo only	0.13 (0.82–1.96)	0.280	5.20 (3.35–8.07)	< 0.001	Ref	
Surgery +chemo	0.44 (0.28–0.68)	< 0.001	1.75 (1.13–2.73)	0.013	0.34 (0.20–0.58)	< 0.001
Gastrointestinal stromal sarcoma (GISS)						
None	Ref					
Surgery only	0.51 (0.14–1.94)	0.324	Ref			
Chemo only	0.18 (0.05–0.68)	0.012	0.35 (0.09-1.40)	0.138	Ref	
Surgery + chemo	0.25 (0.09–0.73)	0.012	0.48 (0.15–1.51)	0.209	1.36 (0.43–4.28)	0.599
Part II: multivariate analysis^a^						
Small bowel cancer						
None	Ref					
Surgery only	0.31 (0.22–0.43)	< 0.001	Ref			
Chemo only	0.70 (0.57–0.86)	0.001	3.04 (2.09–4.42)	< 0.001	Ref	
Surgery + chemo	0.41 (0.30–0.57)	< 0.001	1.48 (1.07–2.04)	0.018	0.47 (0.32–0.67)	< 0.001
Adenocarcinoma						
None	Ref					
Surgery only	0.69 (0.40–1.18)	0.173	Ref			
Chemo only	0.35 (0.27–0.44)	< 0.001	0.57 (0.31–1.02)	0.058	Ref	
Surgery + chemo	0.27 (0.18–0.42)	< 0.001	0.37 (0.22–0.65)	< 0.001	0.67 (0.42–1.06)	0.089
Neuroendocrine carcinoma (NEC)						
None	Ref					
Surgery only	0.24 (0.14–0.43)	< 0.001	Ref			
Chemo only	1.13 (0.68–1.88)	0.633	2.81 (1.41–5.63)	0.003	Ref	
Surgery + chemo	0.39 (0.20–0.73)	0.004	1.48 (0.91–2.41)	0.112	0.52 (0.21–1.31)	0.164
Gastrointestinal stromal sarcoma (GISS)						
None	Ref					
Surgery only	0.31 (0.02–4.71)	0.397	Ref			
Chemo only	0.08 (0.01–0.69)	0.022	0.34 (0.04–2.81)	0.314	Ref	
Surgery + chemo	0.10 (0.01–1.24)	0.073	0.40 (0.08–2.00)	0.262	1.26 (0.21–7.73)	0.804

*CI* confidence interval, *HR* hazard ratio, *Chemo* chemotherapy

^a^Adjusted variables included age, gender, marital status, primary site, histologic grade, T stage, tumor size, N stage, and number of extrahepatic metastatic sites.

## Discussion

The current study described the frequency and prognosis of small bowel cancer patients with liver metastases at their initial diagnosis by using available data from the SEER database. We also explored the predictive indicators for the presence of liver metastases, and sought its optimal treatment modalities based on the survival data, with an attempt to better understand the clinical significance of liver metastases. Since early diagnosis and reasonable treatment may improve overall survival and quality of life, it is of great significance to investigate small bowel cancer patients who had liver metastases in a large independent cohort.

In our study, we totally identified 8831 small bowel cancer patients, of which neuroendocrine tumors (61.2%) were the most common histologic type. This data was not consistent with previous studies that considered adenocarcinomas as the most commonly occurring malignant neoplasms in the small bowel [1, 18]. However, based on the data of small bowel malignancies from National Cancer Data Base (NCDB, 1985–2005), the proportion of carcinoid tumors, which consists mainly of NET and NEC, increased significantly from 27.5% to 44.3%, whereas the proportion of adenocarcinomas decreased from 42.1% to 32.6% [19]. It was also reported that carcinoid tumors surpassed adenocarcinomas as the most common small bowel tumor [19]. In addition, the incidence of carcinoid tumors increased from 2.1 to 9.3 per million (percent change: 340.5%; annual percentage change: 3.6%) from 1973 to 2004, whereas the incidence of adenocarcinoma increased with less pronounced. Despite the existence of potential selection bias, we hold the opinion that the proportion of neuroendocrine carcinomas would have increased recently. We also found that 25.9% of patients were diagnosed with synchronous metastatic disease among which 63.9% initially presented with liver metastases. Specifically, distant metastases were occurred in 20.6% of patients with adenocarcinoma, 16.0% of NETs, and 9.5% of GISTs. These result differed slightly from the previous published studies wherein distant metastases were noted on presentation in 24.0% of adenocarcinoma, 15.6% of NETs, and 9.5% of NETs [19].

The risk factors for the occurrence of liver metastases at initial diagnosis were identified using multivariate logistic regression in order to distinguish patients at increased risk for liver metastases. We found that patients were easier to have liver metastases when they had risk factors as follow: tumor located in the lower part of small bowel, poorer histological grade, larger tumor size, later N staging, and presence of more extrahepatic metastatic sites. However, our study failed to demonstrate that tumors with T4 and N2 stage had higher risk of occurring liver metastases in comparison with T0 and N0, respectively. A similar result was also shown in gastric cancer [20]. This result, to some extent, implied that traditional AJCC TNM staging was not sufficient to predict the presence of liver metastases. As a replacement, our study indicated that the tumor size could effectively predict the occurrence of liver metastases, largely because of the reason that large tumor had more chance to occur lymphatic dissemination, hematogenous dissemination, and serosal invasion. In addition, our results also demonstrated that demographic characteristics, such as age, race and insurance status, were not risk factors for the liver metastases, which was not in accordance with previous studies concentrating on gastric cancer [20–22]. Moreover, based on the multivariate logistic regression model, ROC curves incorporating seven independent risk factors showed the best predictive value, with an AUC value equal to 0.75, which were significantly higher than single predictors ranging from 0.44 to 0.65. This result indicated that our combined indicators had an acceptable performance to predict the occurrence of liver metastases, and could be used to distinguish patients who need further examination, like MRI or PET-CT.

Consistent with previous study [23, 24], our result demonstrated that patients with liver metastases had a poor survival when compared with those without liver metastases. Then, multivariate Cox analyses were performed to determine prognostic factors for small bowel cancer patients with different histological types who had liver metastases. The result showed that older age, higher histological grade, or more extrahepatic metastatic site had negative impact on prognosis of patients with NETs. In addition, small bowel cancer patients with tumors occurred in duodenum tend to have the worst prognosis both in adenocarcinoma and NETs set. However, our study found no significant prognostic factor for patients with GISTs. This result was inconsistent with previous study indicating that the prognosis of small intestinal GISTs depends upon tumors size and site of origin [25], which might owe to this subset with 92 patients only.

Due to the rarity and heterogeneity of small bowel cancer, few studies have been done to investigate the optimal treatment modalities. The management of small bowel cancer, for a long time, was based on the treatment strategy for large bowel cancer [26]. However, a number of anatomical and molecular differences strongly suggest the necessity to update the clinical management of small bowel cancer [27]. Recently, surgery is the primary therapy for most small bowel tumors presenting as locoregional disease [5, 19], which have been reported to significantly prolong patients’ survival [28]. Nevertheless, for patients with metastatic small bowel cancer, the exact role of curative-intent surgery still remains unclear. In current study, the survival benefit of different treatment modalities toward patients with liver metastases was compared based on the multivariate Cox model. For adenocarcinoma, albeit no survival benefit were shown in surgical treatment alone, our result indicated that combination of surgery and systemic chemotherapy could dramatically prolonged patient’s survival when compared with no treatment. In addition, consistent with previous studies [16, 29, 30], our data also found the survival advantage of palliative chemotherapy alone. Since the high morbidity from obstruction (vomiting and poor nutrition) and bleeding caused by small bowel adenocarcinomas, it is our belief that combination of palliative surgery and systemic chemotherapy might improve prognosis and quality of life. For small bowel neuroendocrine tumors (NETs), available evidence and guidelines unanimously recommend resection of a primary tumor site and liver metastatic foci when feasible [31–34], largely because of the intermittent small bowel obstruction or even ischemia caused by neuroendocrine tumor-associated desmoplastic reaction and fibrosis [35]. Similarly, our result also demonstrated that surgery alone served as the best therapeutic option in terms of survival outcome. Furthermore, we also demonstrated that patients with gastrointestinal stromal tumors (GISTs) could only benefit from chemotherapy alone when liver metastases occurred. It was reported that adjuvant therapy of tyrosine kinase receptor inhibitor should be recommended toward those GIST patients with metastatic disease [36]. Taken together, these data showed that the optimal treatment modalities varies across different histologic types of small bowel cancer.

Inevitably, the current study has some limitations. Firstly, more detail information, such as comorbidities, performance status, the size and location of liver metastases, adjuvant therapy in terms of dose, mitotic rate, and intra-operative tumor capsule rupture, were lacking in the SEER program. Secondly, a large number of patients with incomplete or unqualified information were exclude in the study, which may induce potential selection bias. We believe that all the observed results in our study should be prospectively validated.

## Conclusion

In summary, this study provided investigation of the frequency for liver metastases of small bowel cancer at initial diagnosis. Primary tumor presented with lower part of small intestine, poor tumor grade, larger tumor size, later N staging, and presence of more extrahepatic metastatic sites had increased propensity of developing liver metastases. The combined predictor had a good ability to predict the presence of liver metastases. Patients with liver metastases had significant poorer survival than those without liver metastases. In addition, combination of surgery and chemotherapy conferred the optimal survival for patients with adenocarcinoma, while the optimal treatment options for NEC and GISS seemed to be surgery alone and chemotherapy alone, respectively.

## Supplementary information

**Additional file 1: Table S1**. Clinical characteristics of small bowel cancer patients with or without liver metastases at diagnosis.

**Additional file 2: Table S2**. Univariate analysis for overall survival (OS) and cancer-specific survival (CSS) among patients with small bowel adenocarcinoma who had liver metastasis.

**Additional file 3: Table S3**. Univariate analysis for overall survival (OS) and cancer-specific survival (CSS) among patients with small bowel neuroendocrine tumors (NETs) who had liver metastasis.

**Additional file 4: Table S4**. Univariate analysis for overall survival (OS) and cancer-specific survival (CSS) among patients with small bowel gastrointestinal stromal tumor (GIST) who had liver metastasis.

## Data Availability

The datasets generated and/or analysed during the current study are available in the Surveillance, Epidemiology, and End Results Program repository, https://seer.cancer.gov/data/.

## Abbreviations

SEER: Surveillance, Epidemiology, and End Results
NETs: Neuroendocrine tumors
GIST: Gastrointestinal stromal tumor
OS: Overall survival
CSS: Cancer-specific survival
OR: Odd ratio
HR: Hazard ratio
ROC: Receiver operating characteristic curve
AUC: Area under the curve
