# Supplementary material for: Frequency, prognosis and treatment modalities of newly diagnosed small bowel cancer with liver metastases
Source: BMC Gastroenterol. 2020 Oct 15;20:342. doi: 10.1186/s12876-020-01487-6 (PMC7558693; doi:10.1186/s12876-020-01487-6)
Supplement: Supplementary file 1 — Additional file 1: Table S1. Clinical characteristics of small bowel cancer patients with or without liver metastases at diagnosis. [file 12876_2020_1487_MOESM1_ESM.docx]

Table S1. Clinical characteristics of small bowel cancer patients with or without liver metastases at diagnosis.

| Variables | Total, n (%)  N = 8831 | Without Liver Metastases, n (%)  N = 7370 | With Liver  Metastases, n (%)  N = 1461 | P value |
| --- | --- | --- | --- | --- |
| Year at diagnosis |  |  |  | 0.665 |
| 2010-2011 | 2747 (31.1) | 2303 (31.2) | 444 (30.4) |  |
| 2012-2013 | 2932 (33.2) | 2451 (33.3) | 481 (32.9) |  |
| 2014-2015 | 3152 (35.7) | 2616 (35.5) | 536 (36.7) |  |
| Age |  |  |  |  |
| <40 | 369 (4.2) | 305 (4.1) | 64 (4.4) | 0.360 |
| 40-59 | 2998 (33.9) | 2489 (33.8) | 509 (34.8) |  |
| 60-79 | 4371 (49.5) | 3644 (49.4) | 727 (49.8) |  |
| ≥80 | 1093 (12.4) | 932 (12.6) | 161 (11.0) |  |
| Race |  |  |  | 0.004 |
| Black | 1485 (16.8) | 1244 (16.9) | 241 (16.5) |  |
| White | 6791 (76.9) | 5643 (76.6) | 1148 (78.6) |  |
| Others^a^ | 465 (5.3) | 396 (5.4) | 69 (4.7) |  |
| Unknown | 90 (1.0) | 87 (1.2) | 3 (0.2) |  |
| Gender |  |  |  | 0.501 |
| Male | 4553 (51.6) | 3788 (51.4) | 765 (52.4) |  |
| Female | 4278 (48.4) | 3582 (48.6) | 696 (47.6) |  |
| Insurance status |  |  |  | 0.014 |
| No | 263 (3.0) | 211 (2.9) | 52 (3.6) |  |
| Yes | 8373 (94.8) | 6983 (94.7) | 1390 (95.1) |  |
| Unknown | 195 (2.2) | 176 (2.4) | 19 (1.3) |  |
| Marital status |  |  |  | 0.245 |
| Unmarried | 3222 (36.5) | 2693 (36.5) | 529 (36.2) |  |
| Married | 5089 (57.6) | 4230 (57.4) | 859 (58.8) |  |
| Unknown | 520 (5.9) | 447 (6.1) | 73 (5.0) |  |
| Primary site |  |  |  | <0.001 |
| Duodenum | 3201 (36.2) | 2763 (37.5) | 438 (30.0) |  |
| Jejunum | 848 (9.6) | 737 (10.0) | 111 (7.6) |  |
| Ileum | 2572 (29.1) | 2114 (28.7) | 458 (31.3) |  |
| Other site^b^ | 141 (1.6) | 125 (1.7) | 16 (1.1) |  |
| Unknown | 2069 (23.4) | 1631 (22.1) | 438 (30.0) |  |
| Grade |  |  |  | <0.001 |
| I | 3704 (41.9) | 3240 (44.0) | 464 (31.8) |  |
| II | 1908 (21.6) | 1618 (22.0) | 290 (19.8) |  |
| III | 849 (9.6) | 663 (9.0) | 186 (12.7) |  |
| IV | 125 (1.4) | 98 (1.3) | 27 (1.8) |  |
| Unknown | 2245 (25.4) | 1751 (23.8) | 494 (33.8) |  |
| Histologic type |  |  |  | <0.001 |
| Adenocarcinoma | 2457 (27.8) | 1951 (26.5) | 506 (34.6) |  |
| NEC | 5406 (61.2) | 4543 (61.6) | 863 (59.1) |  |
| GISS | 968 (11.0) | 876 (11.9) | 92 (6.3) |  |
| T stage |  |  |  | <0.001 |
| T1 | 1382 (15.6) | 1284 (17.4) | 98 (6.7) |  |
| T2 | 1306 (14.8) | 1192 (16.2) | 114 (7.8) |  |
| T3 | 2767 (31.3) | 2349 (31.9) | 418 (28.6) |  |
| T4 | 2128 (24.1) | 1701 (23.1) | 427 (29.2) |  |
| Unknown | 1248 (14.1) | 844 (11.5) | 404 (27.7) |  |
| Tumor size, cm |  |  |  | <0.001 |
| 0-1 | 1518 (17.2) | 1466 (19.9) | 52 (3.6) |  |
| 1-2 | 1897 (21.5) | 1630 (22.1) | 267 (18.3) |  |
| 2-5 | 2523 (28.6) | 2035 (27.6) | 488 (33.4) |  |
| >5 | 1325 (15.0) | 1149 (15.6) | 176 (12.0) |  |
| Unknown | 1568 (17.8) | 1090 (14.8) | 478 (32.7) |  |
| N stage |  |  |  | <0.001 |
| N0 | 4732 (53.6) | 4201 (57.0) | 531 (36.3) |  |
| N1 | 3430 (38.8) | 2702 (36.7) | 728 (49.8) |  |
| N2 | 315 (3.6) | 264 (3.6) | 51 (3.5) |  |
| Unknown | 354 (4.0) | 203 (2.8) | 151 (10.3) |  |
| Extrahepatic metastatic sites to bone, lung, and brain, No. | | | | <0.001 |
| 0 | 8488 (96.1) | 7233 (98.1) | 1255 (85.9) |  |
| 1 | 243 (2.8) | 101 (1.4) | 142 (9.7) |  |
| 2 | 16 (0.2) | 7 (0.1) | 9 (0.6) |  |
| Unknown | 84 (1.0) | 29 (0.4) | 55 (3.8) |  |
| Surgery |  |  |  | <0.001 |
| No | 1865 (21.1) | 1191 (16.2) | 674 (46.1) |  |
| Yes | 6935 (78.5) | 6150 (83.4) | 785 (53.7) |  |
| Unknown | 31 (0.4) | 29 (0.4) | 2 (0.1) |  |
| Radiotherapy |  |  |  | <0.001 |
| No | 8563 (97.0) | 7176 (97.4) | 1387 (94.9) |  |
| Yes | 268 (3.0) | 194 (2.6) | 74 (5.1) |  |
| Chemotherapy |  |  |  | <0.001 |
| No | 6820 (77.2) | 5902 (80.1) | 918 (62.8) |  |
| Yes | 2011 | 1468 (19.9) | 543 (37.2) |  |

Abbreviations:

NEC: neuroendocrine carcinoma; GISS: gastrointestinal stromal sarcoma

^a^ including Asian and American Indians;

^b^ including meckels diverticulum, and overlapping lesion of small intestine;
