# Supplementary material for: Frequency, prognosis and treatment modalities of newly diagnosed small bowel cancer with liver metastases
Source: BMC Gastroenterol. 2020 Oct 15;20:342. doi: 10.1186/s12876-020-01487-6 (PMC7558693; doi:10.1186/s12876-020-01487-6)
Supplement: Supplementary file 2 — Additional file 2: Table S2. Univariate analysis for overall survival (OS) and cancer-specific survival (CSS) among patients with small bowel adenocarcinoma who had liver metastasis. [file 12876_2020_1487_MOESM2_ESM.docx]

Table S2. Univariate analysis for overall survival (OS) and cancer-specific survival (CSS) among patients with small bowel adenocarcinoma who had liver metastasis.

|  | OS | |  | CSS | |
| --- | --- | --- | --- | --- | --- |
| Variables | HR (95% CI) | P value |  | HR (95% CI) | P value |
| Age |  |  |  |  |  |
| <40 | Reference |  |  | Reference |  |
| 40-59 | 0.92 (0.56-1.52) | 0.745 |  | 0.89 (0.54-1.46) | 0.641 |
| 60-79 | 1.14 (0.71-1.85) | 0.582 |  | 1.02 (0.63-1.65) | 0.582 |
| ≥80 | 1.88 (1.13-3.12) | 0.016 |  | 1.74 (1.04-2.91) | 0.034 |
| Race |  |  |  |  |  |
| Black | Reference |  |  | Reference |  |
| White | 1.28 (1.03-1.60) | 0.029 |  | 1.28 (1.02-1.62) | 0.036 |
| Others^a^ | 1.10 (0.74-1.66) | 0.635 |  | 1.21 (0.80-1.82) | 0.366 |
| Gender |  |  |  |  |  |
| Male | Reference |  |  | Reference |  |
| Female | 0.99 (0.82-1.19) | 0.904 |  | 0.97 (0.80-1.18) | 0.768 |
| Insurance status |  |  |  |  |  |
| No | Reference |  |  | Reference |  |
| Yes | 1.24 (0.73-2.11) | 0.435 |  | 1.14 (0.67-1.95) | 0.631 |
| Unknown | 2.22 (0.80-6.17) | 0.128 |  | 1.82 (0.60-5.54) | 0.293 |
| Marital status |  |  |  |  |  |
| Unmarried | Reference |  |  | Reference |  |
| Married | 0.88 (0.73-1.07) | 0.196 |  | 0.93 (0.76-1.13) | 0.452 |
| Unknown | 1.04 (0.68-1.60) | 0.859 |  | 1.01 (0.64-1.60) | 0.967 |
| Primary site |  |  |  |  |  |
| Duodenum | Reference |  |  | Reference |  |
| Jejunum | 0.56 (0.41-0.77) | <0.001 |  | 0.54 (0.38-0.75) | <0.001 |
| Ileum | 0.78 (0.53-1.14) | 0.201 |  | 0.82 (0.56-1.21) | 0.312 |
| Other site^b^ | 0.39 (1.15-1.06) | 0.064 |  | 0.42 (0.16-1.14) | 0.089 |
| Unknown | 0.89 (0.67-1.17) | 0.397 |  | 0.89 (0.67-1.19) | 0.424 |
| Grade |  |  |  |  |  |
| I | Reference |  |  | Reference |  |
| II | 0.70 (0.41-1.22) | 0.212 |  | 0.65 (0.37-1.13) | 0.130 |
| III | 1.10 (0.63-1.90) | 0.740 |  | 1.00 (0.58-1.75) | 0.975 |
| IV | 1.07 (0.31-3.72) | 0.919 |  | 1.07 (0.31-3.73) | 0.914 |
| Unknown | 0.98 (0.57-1.69) | 0.947 |  | 0.88 (0.51-1.52) | 0.641 |
| T stage |  |  |  |  |  |
| T1 | Reference |  |  | Reference |  |
| T2 | 1.03 (0.48-2.24) | 0.935 |  | 1.16 (0.53-2.52) | 0.710 |
| T3 | 0.60 (0.42-0.85) | 0.004 |  | 0.59 (0.41-0.86) | 0.005 |
| T4 | 0.83 (0.63-1.10) | 0.190 |  | 0.84 (0.63-1.12) | 0.233 |
| Unknown | 1.17 (0.90-1.53) | 0.242 |  | 1.23 (0.93-1.62) | 0.144 |
| Tumor size, cm |  |  |  |  |  |
| 0-1 | Reference |  |  | Reference |  |
| 1-2 | 1.12 (0.47-2.65) | 0.795 |  | 1.12 (0.44-2.84) | 0.816 |
| 2-5 | 1.24 (0.58-2.65) | 0.580 |  | 1.36 (0.60-3.08) | 0.468 |
| >5 | 1.52 (0.69-3.34) | 0.295 |  | 1.70 (0.73-3.96) | 0.217 |
| Unknown | 1.67 (0.79-3.55) | 0.180 |  | 1.79 (0.80-4.03) | 0.159 |
| N stage |  |  |  |  |  |
| N0 | Reference |  |  | Reference |  |
| N1 | 0.77 (0.63-0.96) | 0.019 |  | 0.78 (0.62-0.97) | 0.029 |
| N2 | 0.69 (0.50-0.96) | 0.025 |  | 0.71 (0.51-0.98) | 0.039 |
| Unknown | 0.94 (0.73-1.23) | 0.666 |  | 0.92 (0.70-1.22) | 0.576 |
| Extrahepatic metastatic sites to bone, lung, and brain, No. | | |  |  |  |
| 0 | Reference |  |  | Reference |  |
| 1 | 1.00 (0.79-1.27) | 0.981 |  | 1.03 (0.81-1.32) | 0.789 |
| 2 | 1.32 (0.62-2.79) | 0.471 |  | 1.46 (0.69-3.08) | 0.328 |
| Unknown | 1.26 (0.86-1.85) | 0.228 |  | 1.24 (0.83-1.86) | 0.287 |

Abbreviations:

CI: confidence interval; HR: Hazard ratio;

^a^ including Asian and American Indians;

^b^ including meckels diverticulum, and overlapping lesion of small intestine;
