# Supplementary material for: Frequency, prognosis and treatment modalities of newly diagnosed small bowel cancer with liver metastases
Source: BMC Gastroenterol. 2020 Oct 15;20:342. doi: 10.1186/s12876-020-01487-6 (PMC7558693; doi:10.1186/s12876-020-01487-6)
Supplement: Supplementary file 3 — Additional file 3: Table S3. Univariate analysis for overall survival (OS) and cancer-specific survival (CSS) among patients with small bowel neuroendocrine tumors (NETs) who had liver metastasis. [file 12876_2020_1487_MOESM3_ESM.docx]

Table S3. Univariate analysis for overall survival (OS) and cancer-specific survival (CSS) among patients with small bowel neuroendocrine tumors (NETs) who had liver metastasis.

|  | OS | |  | CSS | |
| --- | --- | --- | --- | --- | --- |
| Variables | HR (95% CI) | P value |  | HR (95% CI) | P value |
| Age |  |  |  |  |  |
| <40 | Reference |  |  | Reference |  |
| 40-59 | 2.60 (0.82-8.28) | 0.106 |  | 3.21 (0.78-13.2) | 0.106 |
| 60-79 | 6.18 (1.97-19.4) | 0.002 |  | 7.59 (1.88-30.7) | 0.004 |
| ≥80 | 10.7 (3.27-34.8) | <0.001 |  | 12.5 (2.97-52.8) | 0.001 |
| Race |  |  |  |  |  |
| Black | Reference |  |  | Reference |  |
| White | 1.32 (0.89-1.94) | 0.166 |  | 1.37 (0.88-2.11) | 0.160 |
| Others^a^ | 0.60 (0.18-1.95) | 0.391 |  | 0.50 (0.12-2.10) | 0.341 |
| Gender |  |  |  |  |  |
| Male | Reference |  |  | Reference |  |
| Female | 0.88 (0.69-1.13) | 0.318 |  | 0.94 (0.71-1.23) | 0.643 |
| Insurance status |  |  |  |  |  |
| No | Reference |  |  | Reference |  |
| Yes | 0.79 (0.40-1.53) | 0.478 |  | 0.64 (0.32-1.25) | 0.190 |
| Unknown | 1.20 (0.40-3.59) | 0.740 |  | 1.19 (0.40-3.56) | 0.753 |
| Marital status |  |  |  |  |  |
| Unmarried | Reference |  |  | Reference |  |
| Married | 0.73 (0.57-0.95) | 0.020 |  | 0.73 (0.55-0.98) | 0.035 |
| Unknown | 0.79 (0.44-1.41) | 0.424 |  | 0.82 (0.43-1.53) | 0.528 |
| Primary site |  |  |  |  |  |
| Duodenum | Reference |  |  | Reference |  |
| Jejunum | 0.24 (0.11-0.54) | 0.001 |  | 0.22 (0.09-0.53) | 0.001 |
| Ileum | 0.29 (0.19-0.43) | <0.001 |  | 0.24 (0.16-0.37) | <0.001 |
| Other site^b^ | 0.38 (0.09-1.57) | 0.179 |  | 0.20 (0.03-1.49) | 0.117 |
| Unknown | 0.52 (0.35-0.77) | 0.001 |  | 0.46 (0.30-0.70) | <0.001 |
| Grade |  |  |  |  |  |
| I | Reference |  |  | Reference |  |
| II | 1.18 (0.77-1.80) | 0.439 |  | 1.17 (0.73-1.88) | 0.514 |
| III | 8.87 (5.16-15.2) | <0.001 |  | 8.78 (4.82-16.0) | <0.001 |
| IV | 6.92 (3.19-15.0) | <0.001 |  | 8.48 (3.88-18.5) | <0.001 |
| Unknown | 2.67 (2.01-3.54) | <0.001 |  | 2.77 (2.03-3.80) | <0.001 |
| T stage |  |  |  |  |  |
| T1 | Reference |  |  | Reference |  |
| T2 | 0.74 (0.22-2.50) | 0.626 |  | 0.59 (0.17-2.02) | 0.398 |
| T3 | 0.87 (0.27-2.74) | 0.805 |  | 0.68 (0.21-2.15) | 0.506 |
| T4 | 1.01 (0.32-3.22) | 0.985 |  | 0.85 (0.27-2.72) | 0.785 |
| Unknown | 1.87 (0.59-5.93) | 0.285 |  | 1.57 (0.50-4.99) | 0.443 |
| Tumor size, cm |  |  |  |  |  |
| 0-1 | Reference |  |  | Reference |  |
| 1-2 | 0.88 (0.42-1.86) | 0.737 |  | 0.61 (0.28-1.31) | 0.202 |
| 2-5 | 0.93 (0.45-1.94) | 0.855 |  | 0.77 (0.37-1.62) | 0.492 |
| >5 | 2.04 (0.90-4.60) | 0.087 |  | 1.74 (0.76-4.00) | 0.192 |
| Unknown | 2.41 (1.17-4.97) | 0.017 |  | 2.09 (1.01-4.31) | 0.047 |
| N stage |  |  |  |  |  |
| N0 | Reference |  |  | Reference |  |
| N1 | 0.58 (0.44-0.76) | <0.001 |  | 0.57 (0.42-0.78) | <0.001 |
| N2 | 0.73 (0.54-0.99) | 0.046 |  | 0.78 (0.54-1.01) | 0.061 |
| Unknown | 1.81 (1.23-2.67) | 0.003 |  | 1.98 (1.30-3.01) | 0.001 |
| Extrahepatic metastatic sites to bone, lung, and brain, No. | | |  |  |  |
| 0 | Reference |  |  | Reference |  |
| 1 | 3.04 (2.03-4.53) | <0.001 |  | 3.47 (2.28-5.28) | <0.001 |
| 2 | 0.95 (0.46-1.97) | 0.876 |  | 0.98 (0.40-2.41) | 0.955 |
| Unknown | 1.15 (0.51-2.59) | 0.735 |  | 1.19 (0.49-2.90) | 0.698 |

Abbreviations:

CI: confidence interval; HR: Hazard ratio;

^a^ including Asian and American Indians;

^b^ including meckels diverticulum, and overlapping lesion of small intestine;
