# Supplementary material for: Frequency, prognosis and treatment modalities of newly diagnosed small bowel cancer with liver metastases
Source: BMC Gastroenterol. 2020 Oct 15;20:342. doi: 10.1186/s12876-020-01487-6 (PMC7558693; doi:10.1186/s12876-020-01487-6)
Supplement: Supplementary file 4 — Additional file 4: Table S4. Univariate analysis for overall survival (OS) and cancer-specific survival (CSS) among patients with small bowel gastrointestinal stromal tumor (GIST) who had liver metastasis. [file 12876_2020_1487_MOESM4_ESM.docx]

Table S4. Univariate analysis for overall survival (OS) and cancer-specific survival (CSS) among patients with small bowel gastrointestinal stromal tumor (GIST) who had liver metastasis.

|  | OS | |  | CSS | |
| --- | --- | --- | --- | --- | --- |
| Variables | HR (95% CI) | P value |  | HR (95% CI) | P value |
| Age |  |  |  |  |  |
| <40 | Reference |  |  | Reference |  |
| 40-59 | 1.27 (0.42-3.82) | 0.676 |  | 1.21 (0.42-3.48) | 0.720 |
| 60-79 | 1.58 (0.55-4.51) | 0.394 |  | 1.82 (0.66-5.02) | 0.249 |
| ≥80 | 3.16 (0.99-10.2) | 0.053 |  | 2.66 (0.85-8.29) | 0.092 |
| Race |  |  |  |  |  |
| Black | Reference |  |  | Reference |  |
| White | 1.11 (0.26-4.68) | 0.891 |  | 0.85 (0.20-3.66) | 0.830 |
| Others^a^ | 1.17 (0.23-6.06) | 0.848 |  | 0.72 (0.12-4.33) | 0.721 |
| Gender |  |  |  |  |  |
| Male | Reference |  |  | Reference |  |
| Female | 0.66 (0.30-1.43) | 0.289 |  | 0.69 (0.29-1.67) | 0.415 |
| Insurance status |  |  |  |  |  |
| No | Reference |  |  | Reference |  |
| Yes | 3.01 (0.41-22.1) | 0.278 |  | 2.19 (0.29-16.3) | 0.443 |
| Marital status |  |  |  |  |  |
| Unmarried | Reference |  |  | Reference |  |
| Married | 0.60 (0.29-1.23) | 0.160 |  | 0.47 (0.21-1.08) | 0.075 |
| Unknown | 0.70 (0.09-5.35) | 0.728 |  | 0.83 (0.11-6.47) | 0.858 |
| Primary site |  |  |  |  |  |
| Duodenum | Reference |  |  | Reference |  |
| Jejunum | 0.55 (0.18-1.65) | 0.285 |  | 0.83 (0.25-2.73) | 0.761 |
| Ileum | 1.33 (0.29-6.18) | 0.713 |  | 1.97 (0.40-9.75) | 0.408 |
| Other site^b^ | 1.33 (0.28-6.25) | 0.717 |  | 2.06 (0.41-10.4) | 0.384 |
| Unknown | 0.85 (0.37-1.98) | 0.710 |  | 0.83 (0.29-2.35) | 0.721 |
| Grade |  |  |  |  |  |
| I | Reference |  |  | Reference |  |
| II | 1.17 (0.17-8.36) | 0.873 |  | 0.60 (0.05-6.66) | 0.679 |
| III | 2.17 (0.42-11.2) | 0.355 |  | 1.34 (0.22-8.02) | 0.750 |
| IV | 2.02 (0.42-9.74) | 0.383 |  | 1.47 (0.28-7.63) | 0.646 |
| Unknown | 0.96 (0.22-4.17) | 0.952 |  | 0.79 (0.18-3.54) | 0.761 |
| T stage |  |  |  |  |  |
| T1 | Reference |  |  | Reference |  |
| T2 | 0.99 (0.38-2.58) | 0.977 |  | 0.99 (0.38-2.58) | 0.977 |
| T3 | 1.04 (0.50-2.17) | 0.911 |  | 1.04 (0.50-2.17) | 0.911 |
| T4 | 1.42 (0.73-2.77) | 0.302 |  | 1.42 (0.73-2.78) | 0.302 |
| Tumor size, cm |  |  |  |  |  |
| 0-1 | Reference |  |  | Reference |  |
| 1-2 | 1.52 (0.32-7.13) | 0.597 |  | 1.33 (0.27-6.45) | 0.725 |
| 2-5 | 1.88 (0.51-6.93) | 0.341 |  | 1.72 (0.46-6.41) | 0.418 |
| >5 | 2.17 (0.58-8.13) | 0.249 |  | 2.01 (0.53-7.64) | 0.303 |
| Unknown | 2.39 (0.67-8.45) | 0.177 |  | 2.44 (0.69-8.66) | 0.167 |
| N stage |  |  |  |  |  |
| N0 | Reference |  |  | Reference |  |
| N1 | 1.52 (0.53-4.35) | 0.438 |  | 2.13 (0.72-6.28) | 0.170 |
| N2 | 3.23 (0.43-24.2) | 0.061 |  | 4.45 (0.58-33.9) | 0.150 |
| Extrahepatic metastatic sites to bone, lung, and brain, No. | | |  |  |  |
| 0 | Reference |  |  | Reference |  |
| 1 | 3.27 (0.76-13.9) | <0.001 |  | 4.63 (1.05-20.4) | 0.043 |
| 2 | 1.62 (0.38-6.88) | 0.511 |  | 1.26 (0.29-5.55) | 0.756 |

Abbreviations:

CI: confidence interval; HR: Hazard ratio;

^a^ including Asian and American Indians;

^b^ including meckels diverticulum, and overlapping lesion of small intestine.
